# Evaluation of Home Medication Reconciliation by Clinical Pharmacists for Adult and Pediatric Cystic Fibrosis Patients

**DOI:** 10.3390/pharmacy6030091

**Published:** 2018-08-23

**Authors:** Jessica M. Louie, Lisa T. Hong, Lisa R. Garavaglia, Denise I. Pinal, Catherine E. O’Brien

**Affiliations:** 1 Department of Pharmacy, University of Utah Health, Salt Lake City, UT 84132, USA; 2 Department of Pharmacy Practice, West Coast University School of Pharmacy, Los Angeles, CA 90004, USA; 3 Department of Pharmacy Practice, Loma Linda University School of Pharmacy, Loma Linda, CA 92354, USA; LHong@llu.edu; 4 Department of Pharmacy, West Virginia University Heathcare, Morgantown, WV 26501, USA; Biondol@wvuhealthcare.com; 5 Department of Pharmacy, Cook Children’s Medical Center, Fort Worth, TX 76104, USA; 6 Department of Pharmacy Practice, University of Texas El Paso School of Pharmacy, El Paso, TX 79968, USA; DenisePI@utep.edu; 7 Department of Pharmacy Practice, University of Arkansas for Medical Sciences, Little Rock, AR 72205, USA; ObrienCatherineE@uams.edu

**Keywords:** cystic fibrosis, medication reconciliation, pharmacist, medication errors, guidelines

## Abstract

Medication reconciliation is an important aspect of a patient’s care process that is ideally performed by clinical pharmacists. Despite literature supporting this process in other patient populations, cystic fibrosis (CF) lacks research in this area. To address this, we designed a retrospective, multi-centered, non-controlled, cross-sectional study at four CF Foundation-accredited centers in the United States to evaluate the medication reconciliation process for adult and pediatric CF patients by documenting the number of home medications reconciled by clinical pharmacists and the number of patients with home medications that did not align with the current CF guidelines published in 2013. There were 105 adult patients and 72 pediatric patients included in the study analysis with a mean number of medications reconciled by clinical pharmacists of 17.4 (standard deviation (SD) 6.7) for adults and 13 (SD 4.6) for pediatric patients. The mean number of discrepancies from guidelines per patient was 1.61 (SD 1.2) for adult patients and 0.63 (SD 0.9) for pediatric patients. Pharmacists play an essential role in identifying and managing medication interactions and further research is necessary to investigate pharmacist impact on medication reconciliation.

## 1. Introduction

Medication reconciliation is an important aspect of a patient’s care process. This process is ideally performed by clinical pharmacists to obtain complete and accurate information from a patient regarding medications and resolve discrepancies or potential medication-related problems [1,2]. Several studies demonstrated that medication reconciliation identifies up to 98.2% of unintentional medication discrepancies [3]. The current literature demonstrates the importance of medication reconciliation in the elderly [4], internal medicine [5,6,7], surgery patients [5], and high-risk patients [8]. To date, no literature exists evaluating the benefits of medication reconciliation specifically in the cystic fibrosis (CF) patient population. The European Cystic Fibrosis Society (ECFS) [9] and United Kingdom (UK) Pharmacy Standards of Care [10] for CF clinical pharmacists state that medication reconciliation is the responsibility of CF clinical pharmacists, including medications used for other disease states, research study medications, and over-the-counter (OTC) medications. More recently, the CF Foundation recommended pharmacists as part of the clinical care team.

In accordance with the ECFS and UK standards of care guidelines for CF clinical pharmacists, the primary objective of this study was to evaluate the medication reconciliation process for adult and pediatric CF patients by documenting the number of home medications reconciled by clinical pharmacists and the number of patients with home medications that did not align with current CF guidelines published in 2013 by the American Journal of Respiratory and Critical Care Medicine [11]. The secondary outcomes included quantifying the percentage of patients receiving commonly prescribed medication classes and the number of severe medication interactions per patient.

## 2. Methods

This study was a retrospective, multi-centered, non-controlled, cross-sectional study at four CF Foundation-accredited centers: University of Utah Health Care, University of Arkansas for Medical Sciences, West Virginia University Healthcare, and Cook Children’s Medical Center. Data was collected from January–December, 2014. Patients were included if they had a CF diagnosis and were either admitted to a hospital or outpatient clinic visit with clinical pharmacist involvement. Patients were excluded if they were younger than six years of age.

Data was collected from the most recent medical visit involving a clinical pharmacist and REDCap was utilized for data entry. Severe medication interactions were documented using Micromedex drug interactions and defined as “Contraindicated” or “Major” in severity. The number of discrepancies from the guidelines per patient was determined by comparing the patient’s individual medication list to the CF Foundation guidelines published in 2013 [11]. Any medication that the patient should have been on based on the diagnosis and comorbidities was treated as a discrepancy and vice versa.

## 3. Results

Table 1 displays the demographic data. The mean number of medications reconciled by clinical pharmacists was 17.4 (standard deviation (SD) 6.7) for adults and 13 (SD 4.6) for pediatric patients. The range of medications reconciled was 4–24 medications for pediatric patients and 5–38 medications for adult patients. The mean number of severe medication interactions were 1.63 (SD 4) per adult patient and 0.57 (SD 2.3) per pediatric patient. The mean number of discrepancies from guidelines per patient was 1.61 (SD 1.2) for adult patients and 0.63 (SD 0.9) for pediatric patients. Table 2 displays the results for the medications documented.

## 4. Discussion

The Joint Commission defines medication reconciliation as “the process of comparing the medications a patient is taking (and should be taking) with newly ordered medications” [1,2]. The purpose of medication reconciliation according to the American Society of Health-System Pharmacists (ASHP) is to obtain and maintain complete and accurate information for a patient regarding medications and to resolve potential problems or discrepancies [1]. ASHP believes that pharmacists play an essential role in medication reconciliation to prevent medication errors and support medication safety [1]. Previous studies regarding medication reconciliation demonstrate that the process identifies up to 98.2% of unintentional medication discrepancies [3]. 

As anticipated with a progressive disease such as CF, adult patients in our retrospective review were on a greater number of medications than the pediatric patients. This higher number of medications was associated with a greater number of medication interactions and discrepancies. In comparison to previous studies, Cornish et al. reported that up to 60% of patients will have at least one discrepancy in their admission medication history when admitted to the hospital [4]. These findings align with our findings regarding discrepancies, with the mean number of discrepancies for adult patients at 1.61 per patient. Furthermore, in a study by Lubowski et al. assessing the effectiveness of medication reconciliation in inpatient medical/surgical units, medication discrepancies were identified at a greater rate in patients who were prescribed an average of eight medications [5]. Although there is an inconsistency in defining polypharmacy in the literature [6], the Centers for Medicare and Medicaid Services medication therapy management (MTM) reimbursement requires a patient to be taking a minimum of 2–8 medications. From our findings, the number of home medications in adult and pediatric CF patients far exceeds the Centers for Medicare and Medicaid Services definition of polypharmacy and supports the findings that discrepancies are identified more often with polypharmacy patients.

Our findings support that pharmacists play a role in identifying and managing medication interactions and discrepancies. As can be expected, adult patients had a higher number of interactions identified compared to pediatric patients. Further research is necessary to investigate pharmacist impact on medication reconciliation. Recommendations for further research include correlating health outcomes with discrepancies, documenting the reason for patient medications not aligning with CF guidelines, and the outcomes of clinical pharmacists intervening for the medication reconciliation findings.

## Figures and Tables

**Table 1:** Demographics.

	Pediatrics (*n* = 72) (SD, N (%))	Adults (*n* = 105) (SD, N (%))
Age (mean)	10.9 ± 3.9	29.8 ± 9.3
Female	41 (57)	49 (46.7)
BMI (kg/m^2^)	18.7 ± 3.7	21.1 ± 2.9
Admissions (mean)	0.9 ± 1.7	2 ± 1.6
Clinic visits (mean)	4.5 ± 1.6	3.4 ± 2

**Table 2:** Documented medications.

	Pediatrics (*n* = 72)	Adults (*n* = 105)
Antibiotics		
Tobramycin (Tobi podhaler)	33%	45%
Tobramycin (TIP)	0%	4%
Aztreonam inhaled	17%	25%
Colistin inhaled	1%	2%
Amikacin inhaled	0%	0%
Ceftazidime inhaled	0%	6%
Vancomycin inhaled	0%	2%
Mucolytics		
Dornase alfa	83%	90%
Hypertonic saline	71%	70%
Dornase alfa and hypertonic saline	65%	63%
Acetylcysteine inhaled	0%	3%
Anti-inflammatory		
Azithromycin oral	54%	72%
Corticosteroid oral	3%	8%
Ibuprofen oral scheduled doses	0%	3%
Bronchodilators		
Albuterol	100%	96%
Formoterol	0%	11%
Salmeterol	0%	7%
Inhaled anticholinergics		
Ipratropium	6%	3%
Tiotropium	0%	1%
Aclidinium	0%	1%
Vitamin D		
Cholecalciferol	51%	58%
Ergocalciferol	3%	15%
Other		
Inhaled corticosteroids	47%	53%
Ivacaftor	13%	9%
Multivitamin	93%	73%
Insulin	8%	30%
Pancreatic enzymes	92%	98%
Leukotriene modifiers	18%	24%
